# Concurrent Weekly versus Three Weekly Cisplatin with Radiotherapy in Locally Advanced Uterine Cervical Carcinoma

**DOI:** 10.31729/jnma.3636

**Published:** 2018-10-31

**Authors:** Md. Rakibul Hasan, Md. Abdul Bari, Sarwar Alam, Guru Sharan Sah

**Affiliations:** 1 Department of Oncology, Banghabandhu Sheikh Mujib Medical University, Dhaka, Bangladesh; 2 Department of Medical Oncology, B. P. Koirala Memorial Cancer Hospital, Bharatpur, Nepal

**Keywords:** *Cisplatin*, *concurrent chemotherapy*, *three weekly*, *uterine cervix*, *weekly*

## Abstract

**Introduction:**

Uterine cervical carcinoma is the commonest form of gynecological malignancy in Bangladesh as well as in South Asia. Outcome of weekly versus three weekly Cisplatin concurrent with External beam radiotherapy followed by intracavitary radiotherapy in locally advanced cervical carcinoma was compared in this study.

**Methods:**

A quasi experimental study was carried out from April 2016 to September 2017. Total eighty patients of uterine cervical cancer were included and received External beam radiotherapy concurrent with either weekly or three weekly Cisplatin followed by High dose rate intracavitary brachytherapy. External beam radiotherapy was given with a dose of 50 Gray (Gy) in conventional fractionation over 5 weeks. Cisplatin 40 mg/m^2^, weekly was given along with radiotherapy to the patients of Arm A (n=40) while the patients of Arm B (n=40) received cisplatin 75 mg/m^2^, 3 weekly along with the radiotherapy. Patients were evaluated weekly during treatment and afterwards up to 6 months.

**Results:**

The mean age of patients were 48±9 years for Arm A and 42±9 years for Arm B. Complete response at 6 months of follow up was observed in 30 (75%) and 36 (90%) patients of Arm A and B respectively. Overall complete response was observed in 66 (82.5%)patients. Common toxicities were nephrological, hematological, skin and bowel related and were managed accordingly. Grade III-IV neutropenia was more in patients of Arm A (42.5%) than B (20%).

**Conclusions:**

This study showed that the radiotherapy concurrent with three weekly Cisplatin is effective and less toxic than weekly Cisplatin in locally advanced cervical carcinoma.

## INTRODUCTION

Almost nine out of ten (87%) uterine cervical cancer deaths occur in the less developed regions.^1^ Carcinoma uterine cervix is the second leading cancer among women in Bangladesh.^2^ One third cervical cancer cases of the world are found in the South Asian region, especially in India, Bangladesh, Nepal and Pakistan.^3^

Concurrent chemoradiotherapy (CCRT) is the cornerstone of management for FIGO Stage IIB-IVA disease.^4^ Meta-analysis of some studies (from 1981 to 2000) on chemoradiation for cervical cancer demonstrates improved local control and overall survival with concurrent Cisplatin based chemoradiotherapy.^5^ Most commonly practiced concurrent schedule is weekly Cisplatin during radiotherapy. But study shows that three weekly Cisplatin is more effective and less toxic than weekly Cisplatin.^6^

The objective of this study is to observe and compare the outcome of treatment given by weekly versus three weekly concurrent Cisplatin chemoradiotherapy in locally advanced uterine cervical carcinoma.

## METHODS

This was a Quasi-Experimental study to compare the treatment outcome between radiotherapy concurrent with weekly versus three weekly Cisplatin in the management of locally advanced carcinoma of uterine cervix. The study was conducted at Department of Oncology, Bangabandhu Sheikh Mujib Medical University (BSMMU) and Department of Radiation Oncology, National Institute of Cancer Research and Hospital (NICRH), Dhaka from April 2016 to September 2017. Ethical approval was taken from the Institutional Review Board (IRB) of BSMMU (No. BSMMU/2016/3657 dated 30-03-2016). Sample size was calculated by following formula:


$$\begin{array}{l} \text{n=}\frac{(\text{p1(1-p1)+p(1-p2))}}{\text{(p1-p2)}}\times (\text{Zα +Zβ)\textasciicircum{}2}\\ \text{p1=66.5\%},\text{p2 = 88 .7\%, Zα =}1\text{.96, Zβ=1}.28 \end{array}$$

According to above formula the sample size was needed to be 68 in each Arm, but a total of 80 patients were collected during the study period due to time constrain. Initially patients were selected purposively who met the set inclusion criteria. Then patients were enrolled in either Arm A or Arm B in alternate manner. Inclusion criteria for this study was, clinically diagnosed and histopathologically proven squamous cell carcinoma of uterine cervix FIGO stage IIB to IVA with ECOG performance status 0–2 and no history of prior chemotherapy, radiotherapy or total hysterectomy. Informed written consent was taken from all participated patients.

Forty patients were included in Arm A and received External beam radiotherapy (EBRT), 50Gray (Gy) in 25 fractions over 5 weeks, concurrent with weekly Cisplatin. Arm B included another 40 patients and had received EBRT, 50Gy in 25 fractions over 5 weeks, concurrent with three weekly Cisplatin. Brachytherapy was started one week after the completion of EBRT. Urinary bladder and rectal doses were monitored.

Cisplatin, 40 mg/m^2^ was given weekly to the patients of Arm A on day 1 with 250 ml 0.9% intravenous normal saline over 1 to 2 hour before radiotherapy. The schedule was repeated weekly for 6 weeks. The patients of Arm B received Cisplatin, 75 mg/m^2^ with 250 ml 0.9% intravenous normal saline on day 1, over 1 to 2 hours before radiotherapy. The schedule was repeated three weekly for three cycles.

Premedication (with Ondansetron, Dexamethason and Ranitidine) was given and at least 1 to 3 liters of 0.9% sodium chloride solution depending on the dose of Cisplatin was given to all the patients for hydration. The patients were also asked to take plenty of fluid before and after Cisplatin infusion.

One week after the completion of concurrent chemoradiotherapy, High dose-rate intracavitary radiotherapy (HDR ICRT) was started. All the patients of both the Arms were treated with HDR ICRT, 7Gy per fraction, 3 fractions in consecutive three weeks with a total dose of 21 Gy to the point A. The total duration of the treatment was within 8 weeks. Every patient was evaluated weekly during radiotherapy, thereafter at week 6, 12 and 6 months after completion of treatment to compare the outcome. During evaluation, tumor response was assessed by clinical examination, pervaginal examination and imaging (USG/CT scan). Response Evaluation Criteria in Solid Tumors (RECIST) criteria v1.1 was followed. Toxicities were recorded as per Radiation Therapy Oncology Group/European Organisation for the Research and Treatment (RTOG/ EORTC) scoring system.

All data was collected in a structured data collection form and finally put on a masterchart. For statistical analysis, Statistical Package for Social Sciences (SPSS) version 21 was used. Intention to treat analysis was done. Baseline characteristics were compared by t-test and Z-test. Chi-square test was used to compare the outcomes. A P value of less than 0.05 was considered as statistically significant.

## RESULTS

**Figure 1.**
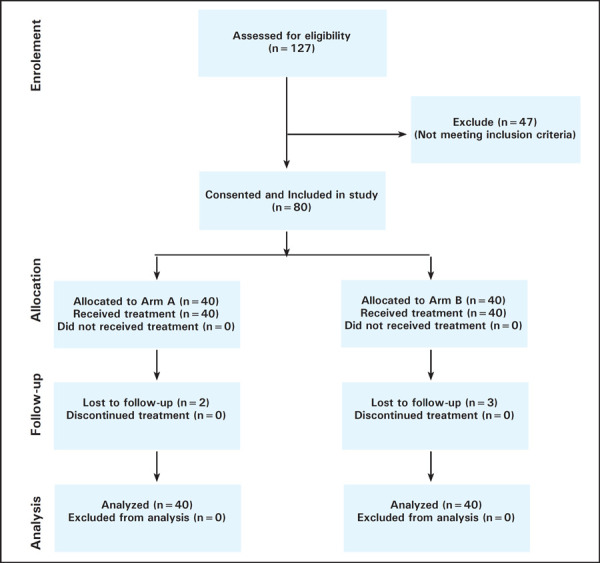
Showing consort flow chart of patients enrolled in the study.

From April 2016 to September 2017, a total of 127 patients were assessed for eligibility and a total of 80 patients were included in this study following the inclusion criteria (Fig.1). Patients were allocated in Arm A and Arm B alternatively. Baseline characteristics of the patient are shown (Table 1). There were 40 patients in Arm A and 40 patients in Arm B. Mean age of the patients in Arm A and B were 48 and 42 years respectively. About 23 (57.5%) and 17 (42.5%) patients of Arm A were in FIGO stage IIB and III respectively, whereas in Arm B it was 21 (52.5%) and 18 (45%). Only one patient of Arm B had stage IVA disease.

**Table 1.** Patient characteristics.

Characteristics	Arm A (n = 35) n (%)	Arm B (n = 35) n (%)		P
Mean Age	48	42		
SD	±9	±9		0.004
Age Range	30–60	25–57		
**ECOG Performance Status**				
0–1	27 (67.5)	23 (52.5)	0.358	
2	13 (32.5)	17 (22.5)	0.358	
FIGO Stage				0.653
III	23 (57.5)	21 (52.5)		0.818
IVA	17 (42.5)	18 (45)		0.313
IVB	0 (0)	1 (2.5)		

In Arm A, complete response was observed in 20 out of 23 patients (87%) and 10 out of 17 patients (58.9%) for Stage IIB and Stage III disease respectively. In Arm B, it was observed in 20 out of 21 patients (95.2%) for Stage IIB and 16 out of 18 patients (88.9%) for Stage III disease. Partial response was seen in 3 of 23 patients (13%) of Stage IIB and 7 of 17 patients (41.2%) of Stage III disease. Surprisingly in Arm B, partial response was observed only in 1of 21 patients (4.8%) and 2 of 18 patients (11.1%) in Stage IIB and Stage III respectively. Only one patient of Arm B was in Stage IVA and showed partial response (Table 1).

**Table 2.** Treatment response after 6 months following completion of treatment according to FIGO stage.

FIGO stage	Response in Arm A (% by stage)	Response in Arm B (% by stage)	P
**Stage IIB**	(n=23)	(n=21)	0.634
	CR^*^:20 (86.96%)	CR:20 (95.23%)	
	PR^†^:3 (13.04%)	PR:1 (04.77%)	
	PD^‡^:0 (0%)	PD:0 (0%)	
**Stage III**	(n = 17)	(n=18)	0.126
	CR:10 (58.83%)	CR:16 (88.89%)	
	PR:7 (41.17%)	PR:2 (11.11%)	
	PD:0 (0%)	PD:0 (0%)	
**Stage IVA**	(n=0)	(n=1)	
	CR:0 (0%)	CR:0 (0%)	
	PR:0 (0%)	PR:1 (100%)	
	PD:0 (0%)	PD:0 (0%)	
**Over All**	(n=40)	(n=40)	0.07
	CR:30 (75%)	CR:36 (90%)	
	PR:10 (25%)	PR:4 (10%)	
	PD:0 (0%)	PD:0 (0%)	

* *CR=Complete Response*

† *PR=Partial Response*

‡ *PD=Progressive Disease*

Toxicities observed during CCRT and ICRT are shown (Table 3, 4). Skin toxicity, vaginal mucositis, bladder, rectum and nephrological toxicities were almost similar in both the Arms, as seen (Table 2). Grade III-IV neutropenia was higher in patients of Arm A, 17 out of 40 (42.5%) compared to Arm B, 8 of 40 (20%) and the difference was statistically significant (Table 3).

**Table 3.** Toxicities observed during concurrent chemotherapy along with radiotherapy.

Toxicity	Arm A (n = 40)	Arm B (n=40)	P
**Skin Reaction**			
Grade 0	16 (40%)	18 (45%)	
Grade I	18 (45%)	17 (42.5%)	0.888
Grade II	6 (15%)	5 (12.5%)	
**Vaginal mucositis**			
Grade 0	24 (60%)	27 (67.5%)	0.73
Grade I	10 (25%)	9 (22.5%)	
Grade II	6 (15%)	4 (10%)	
**Bladder toxicity**			
Grade 0	13 (32.5%)	12 (30%)	0.966
Grade I	17 (42.5%)	18 (45%)	
Grade II	10 (25%)	10 (25%)	
**Nephrologicaltoxicity**			
Grade 0	32 (80%)	28 (70%)	0.494
Grade I	5 (12.5%)	9 (22.5%)	
Grade II	3 (07.5%)	3 (07.5%)	
**Rectal toxicity**			
Grade 0	15 (37.5%)	18 (45%)	
Grade I	12 (30%)	12 (30%)	0.717
Grade II	13 (32.5%)	10 (25%)	

**Table 4.** Neutropenia, thrombocytopenia and neurotoxicity observed during treatment.

Toxicity	Group Arm-A n (%)	Arm- B n (%)	P
**Neutropenia**			
Grade I-II	16 (40.0%)	16 (40.0%)	
Grade III-IV	17 (42.5%)	8 (20.0%)	0.03
**Thrombocytopenia**			
Grade I-II	10 (25.0%)	2 (5.0%)	
Grade III-IV	3 (7.5%)	2 (5.0%)	0.412
**Neuropathy**			
Grade I-II	4 (10.0%)	1 (2.5%)	0.166
Grade III-IV	0 (0.0%)	0 (0.0%)	

## DISCUSSION

Worldwide, uterine cervical carcinoma is one of the leading cause of cancer related morbidity and mortality. Current standard of treatment for locally advanced (FIGO stage III and IVA) cervical cancer is concurrent chemoradiotherapy with weekly Cisplatin followed by ICRT.^7^

Several clinical trials have investigated alternative dose and dosing schedules other than weekly Cisplatin based chemoradiation.^8, 9^ However, most of the studies failed to show any survival benefit. But study done by Ryu et. al. comparing three weekly Cisplatin 75 mg/m^2^ with weekly Cisplatin 40 mg/m^2^ concluded that three weekly Cisplatin is more effective and compliant than weekly cisplatin.^6^ Unfortunately this type of study was not done in less developed countries like Bangladesh.

In this study, diagnosed patients of locally advanced uterine cervical carcinoma (stage IIB to IVA) of squamous cell variety were enrolled. The mean age of patients at diagnosis was 48 years in Arm A and 42 years in Arm B.

Most of the patients had stage IIB disease in both the Arms, of whom 23 (57.5%) and 21 (52.5%) patients were in Arm A and B respectively. About 17 (42.5%) patients of Arm-A and 18 (45.0%) patients of Arm-B were in Stage IIIB disease. Only one patient of stage IVA was in Arm-B.

Follow up at 6 month after completion of treatment, it was observed that 30 (75%) patients showed complete response in Arm A and it was 36 (90%) in Arm B. Statistical analysis revealed that, there was no significant difference (P = 0.07) but mathematically it was seen that the patients of Arm B had better response in respect of tumor size reduction than that of Arm A. This observation correlates with the study done by Ryu et al.^6^

The most prevalent acute toxicities in both the Arms were hematological, nephrological, neurological and gastrointestinal origins. Skin toxicities, vaginal mucositis and rectal toxicities were also observed.

In this study, grade III and IV neutropenia observed during concurrent chemoradiotherapy was higher in weekly than in three weekly Cisplatin Arm. In Arm A, 17 (42.5%) patient experienced Grade III-IV neutropenia, whereas in Arm B, it was observed in 8 (20%) patients. This observation is statistically significant (P = 0.03), as because of shorter recovery time in weekly Cisplatin than that of three weekly schedule. This variation (neutropenia) was also seen in other studies.^10, 11^ However adverse effects were well tolerated and manageable.^11, 12^ From these findings it can be said that three weekly Cisplatin is more compliant and convenient than weekly Cisplatin.

## CONCLUSIONS

Concurrent chemoradiation with three weekly Cisplatin is effective, tolerable, and less toxic than weekly Cisplatin in the treatment of locally advanced carcinoma cervix.
